# Supplementary figures and images for: mRNA induced expression of human angiotensin-converting enzyme 2 in mice for the study of the adaptive immune response to severe acute respiratory syndrome coronavirus 2
Source: PLoS Pathog. 2020 Dec 16;16(12):e1009163. doi: 10.1371/journal.ppat.1009163 (PMC7773324; doi:10.1371/journal.ppat.1009163)

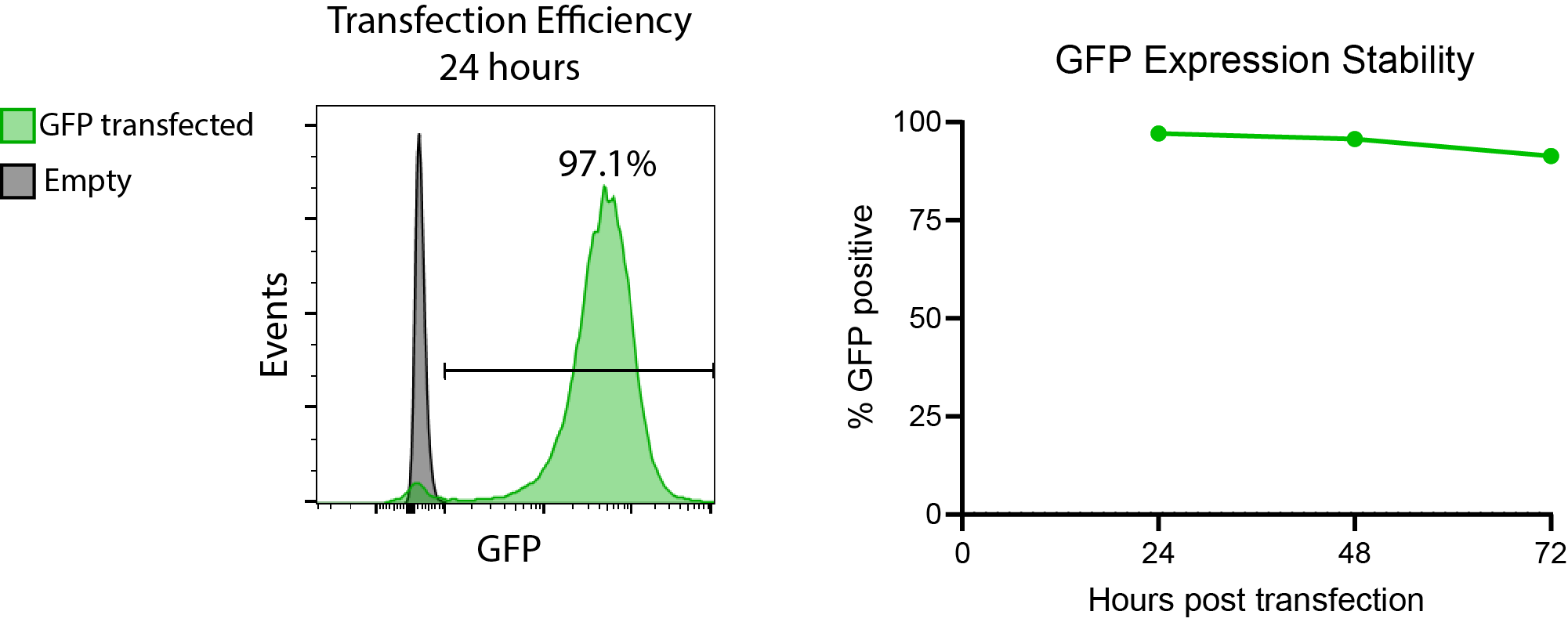

Supplement: S1 Fig — 2x10^6^ Murine 3T3 cells were transfected with 2 μg of either hACE2 or GFP mRNA and plated at a density of 5x10^6^ cells in each well of a 6 well dish. At 24, 48, and 72 hours GFP expression was evaluated by flow cytometry compared to untransfected cells. (TIF) [file ppat.1009163.s001.tif]

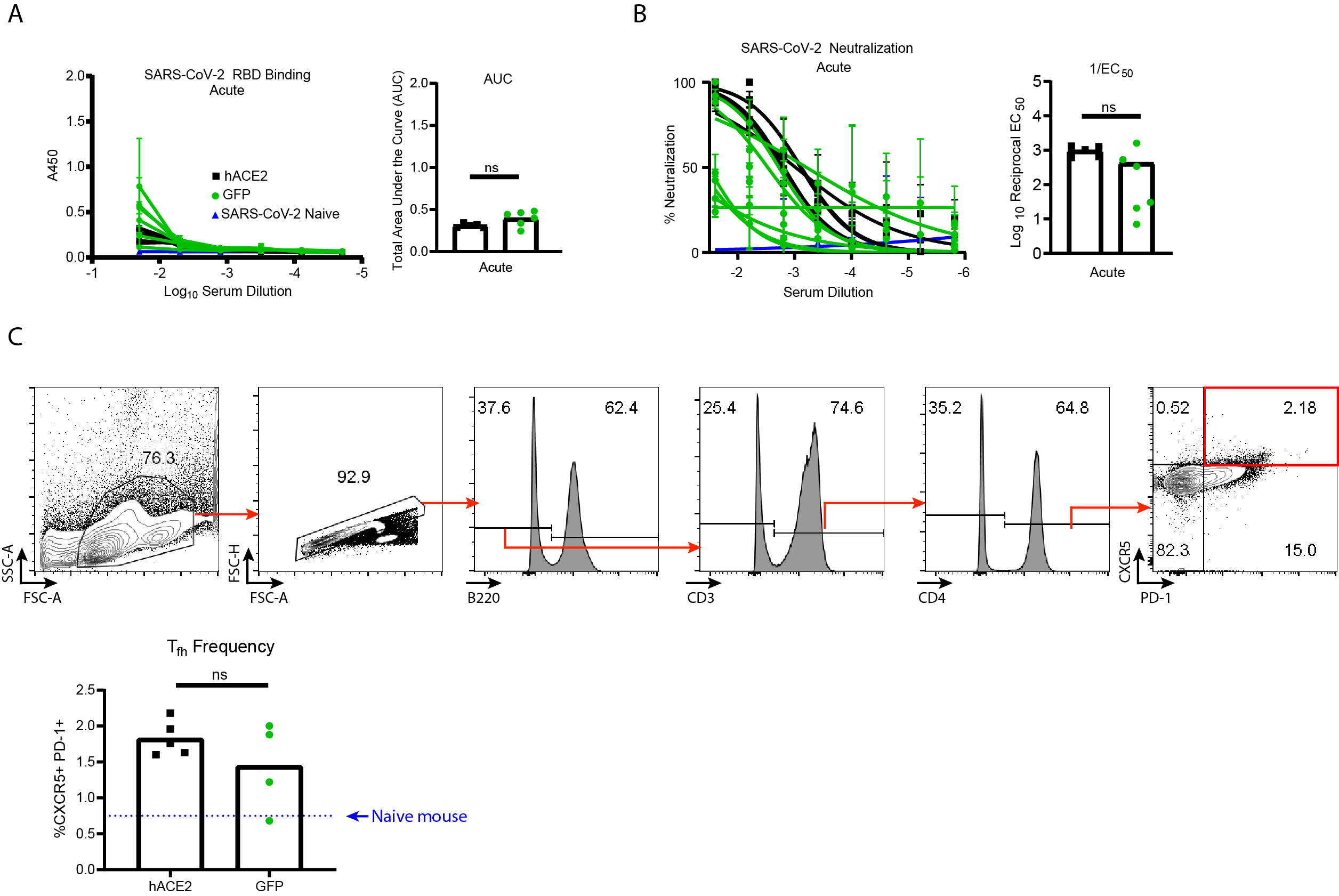

Supplement: S2 Fig — (A) Spike receptor binding domain ELISA. Recombinant SARS-CoV-2 spike RBD protein was used to coat an immunosorbent plate. Serum from transfected and infected mice was serially diluted and used to determine RBD binding potential by absorbance at 450nm with increasing serial dilution and area under the curve calculation. (B) Neutralization potential of polyclonal sera. Serum from transfected and SARS-CoV-2 infected mice was serially diluted and incubated with ~100 focus forming units of SARS-CoV-2 to allow complexes to form. Virus-serum complexes were then overlaid on a Vero-WHO monolayer and allowed to infect for 24 hours, at which point the plates were fixed and developed (see materials and methods). Neutralization was determined by enumerating a reduction in infectious particles with increased serum concentration and determining the EC50. (C) Tfh gating strategy and frequency. Splenocytes from SARS-CoV-2 infected mice were harvested 5 days post boost and were incubated in Fc block in PBS for 1 hour at 4 degrees Celsius. The cells were then washed with PBS and stained for CXCR5, CD62L, CD8, CD4, PD-1, CD3, B220, and CD4 before being washed with PBS and run on an Attune focusing flow cytometer. Tfh cells were defined as lymphocytes based on forward and side scatter, singlets, B220 negative, CD3 positive, CD4 positive, PD-1 and CXCR5 high. Statistical significance was determined by Mann-Whitney test. (TIF) [file ppat.1009163.s002.tif]

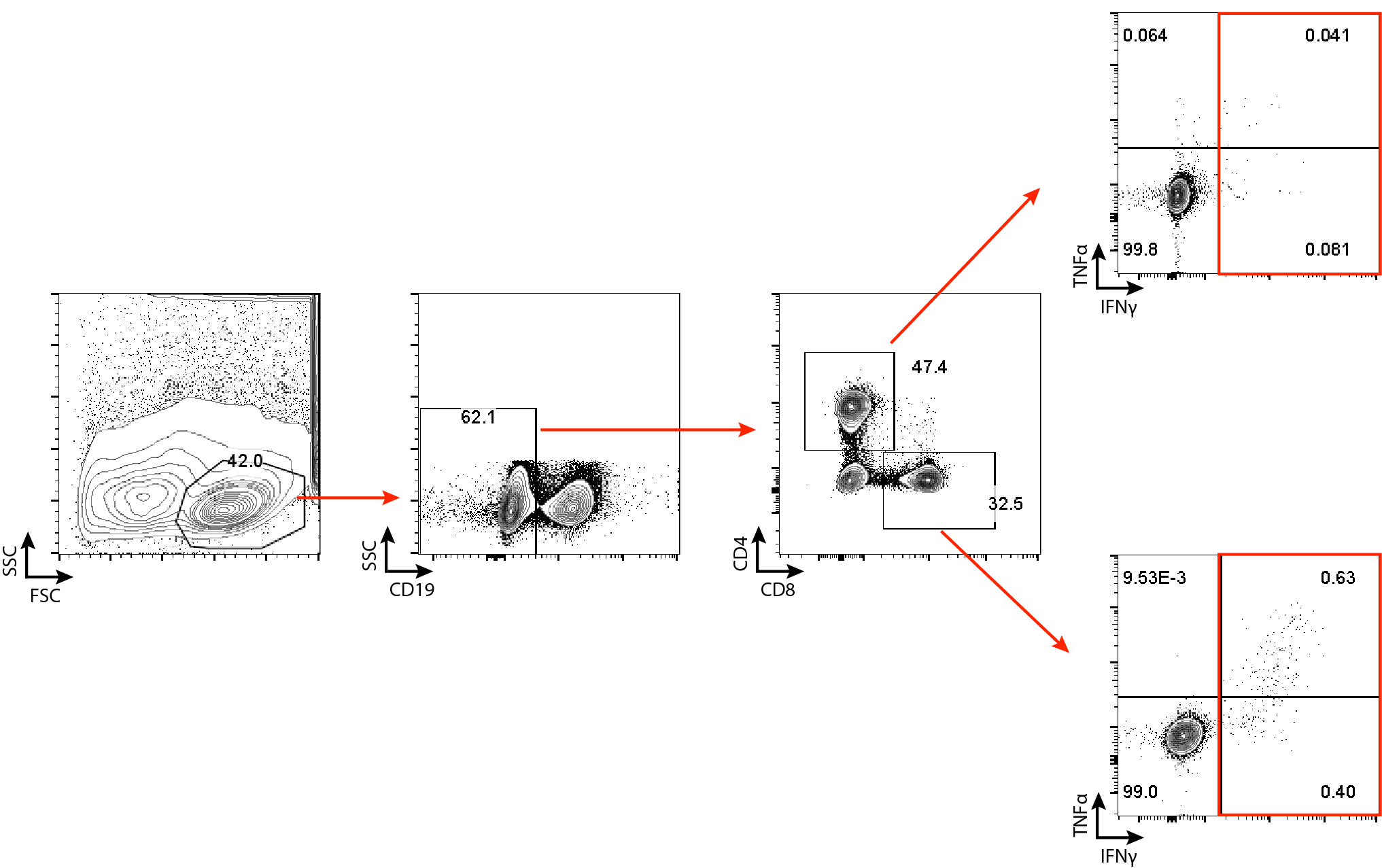

Supplement: S3 Fig — T cells were defined by a lymphocyte gate based on size and granularity and CD19 negative. T cells were further classified as CD8+ or CD4+ T cells by staining CD8+/CD4- or CD4+/CD8- respectively. Antigen responsive T cells were defined by IFN-γ expression. (TIF) [file ppat.1009163.s003.tif]

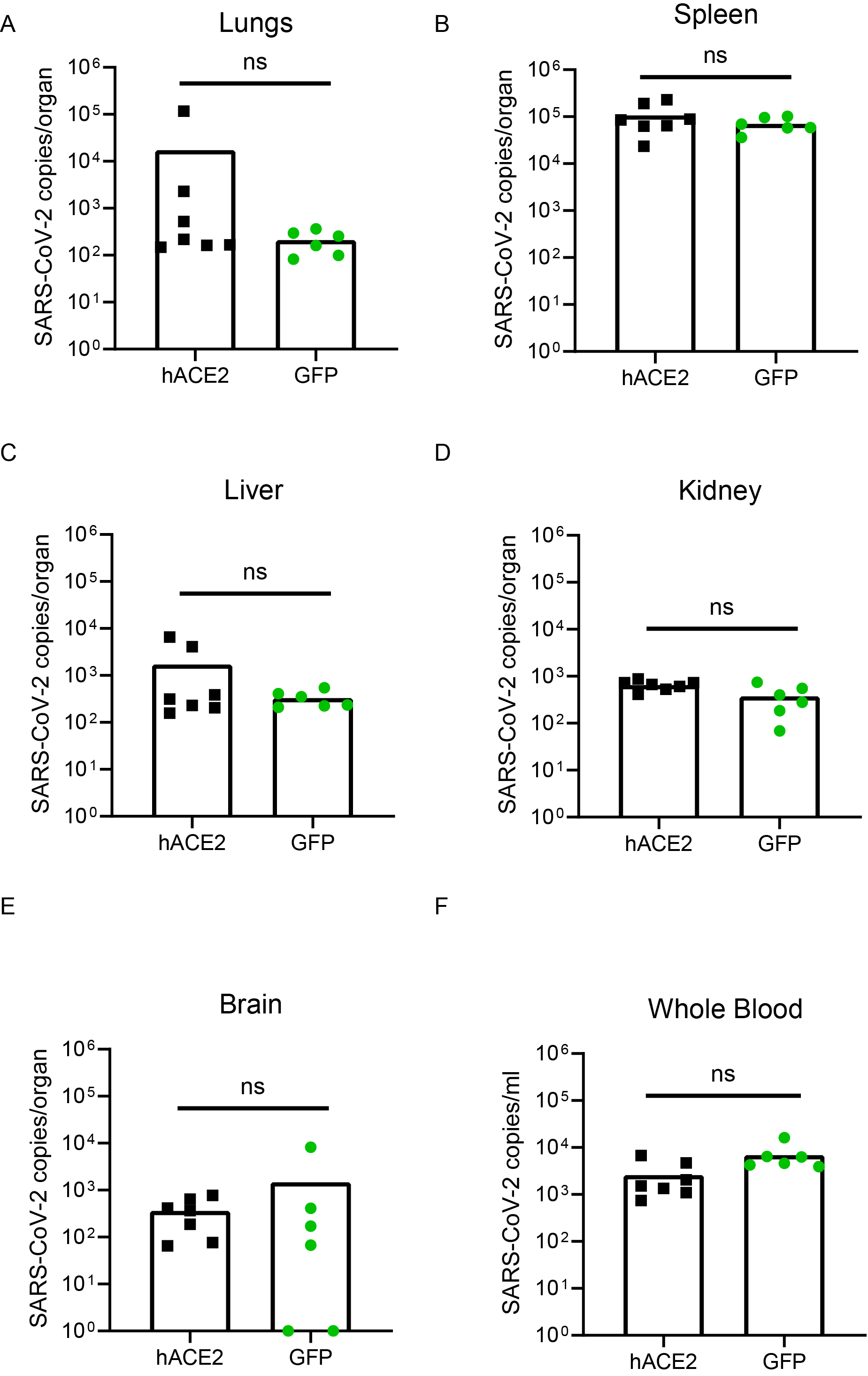

Supplement: S4 Fig — 24 hours following transfections, mice were infected with 5x10^4^ focus forming units (FFU) of SARS-CoV-2 via IV and IN combination route (100 μl and 20 μl, respectively). n = 6 GFP and n = 7 hACE2 were used to quantify viral burden at 3 days post infection in the lungs (A), spleen (B), liver (C), kidney (D), brain (E), and whole blood (F) by qRT-PCR. (TIF) [file ppat.1009163.s004.tif]

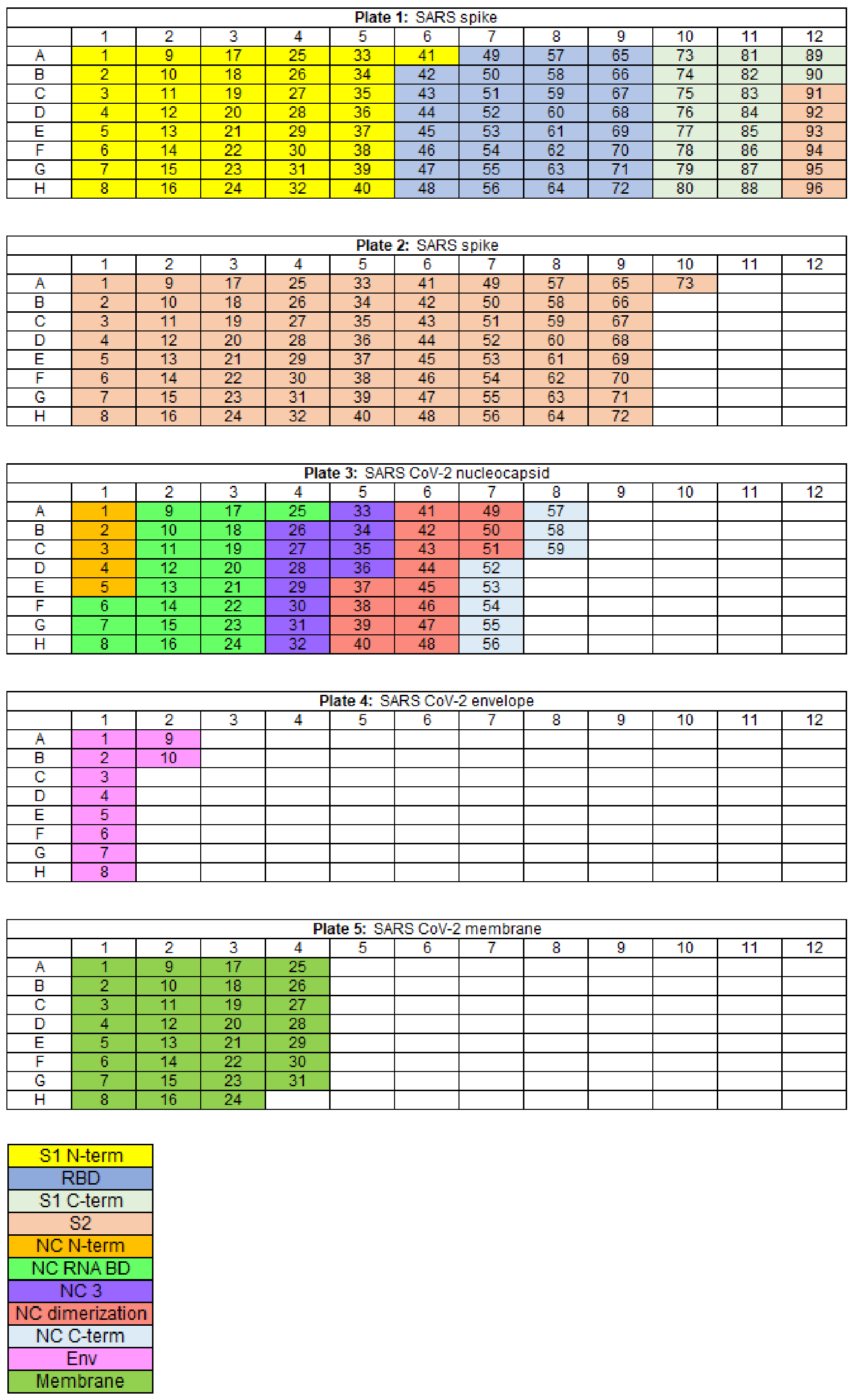

Supplement: S5 Fig — Peptide libraries spanning the SARS-CoV or SARS-CoV-2 structural proteins were obtained from BEI (S1 Table). Every 12-18-mer peptide came in a lyophilized vial and was reconstituted in 90% DMSO to 10 mg/ml and oriented in a 96 well plate format. Subsets of peptides were consolidated to form 11 peptide pools containing various regions or predicted subdomains of each protein (N-terminal region of S1, receptor binding domain, C-terminal region of S1, S2, N-terminal region of nucleocapsid, RNA binding domain of nucleocapsid, nucleocapsid group 3, dimerization domain of nucleocapsid, C- terminal region of nucleocapsid, envelope, and membrane). To aid in identification, peptide pools of 1–5 peptides were also made consisting of the identical well of each plate (e.g. all A1 wells were pooled). (TIF) [file ppat.1009163.s005.tif]

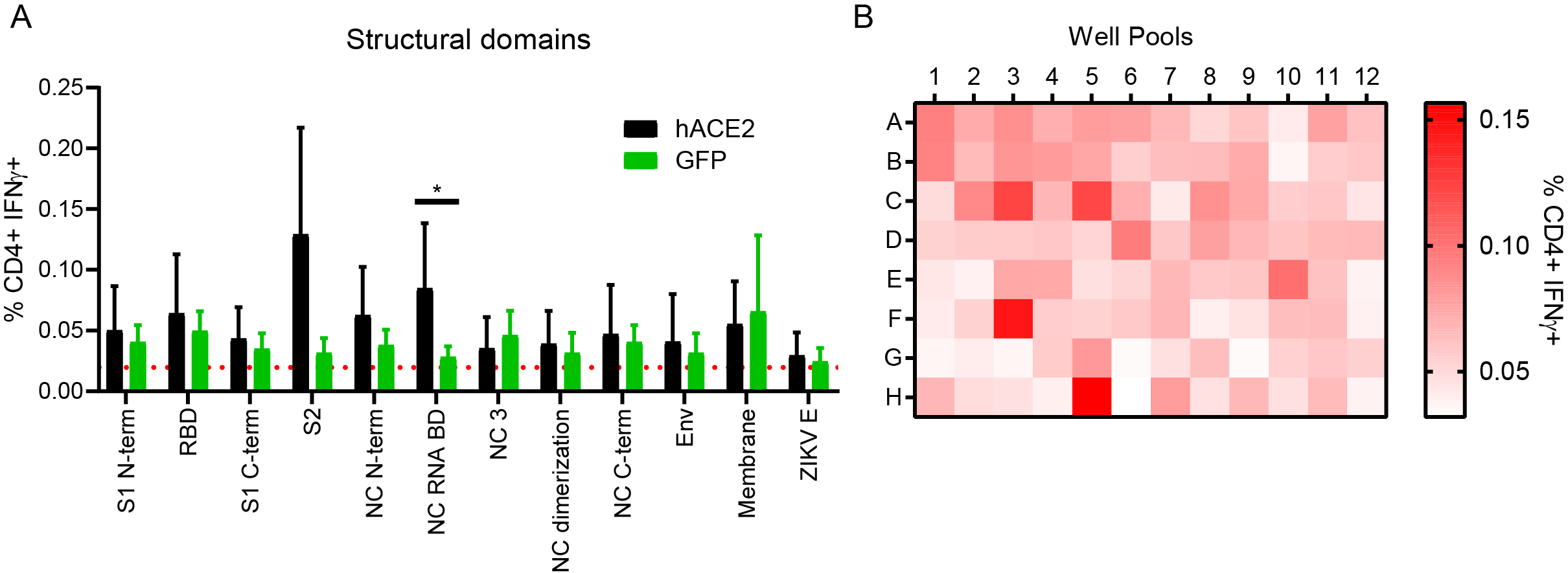

Supplement: S6 Fig — A) CD4+ T cell responses to pooled peptide domains. Each peptide library was demarcated into peptides contained in functional domains of each protein and peptides contained in each domain were pooled into equimolar pools (11 total pools). 5 days post boosted infection with SARS-CoV-2 following transfection with either hACE2 or GFP mRNA, splenocytes were harvested and stimulated for 6 hours with each domain peptide pool in the presence of brefeldin A. After stimulation, cells were stained for flow cytometry to evaluate the frequency of responsive CD4+ T cells by IFN-γ expression. (B) CD4+ T cell responses to smaller “well” peptide pools. Each library was incorporated into multiple 96-well plate formats (S5 Fig). Within the same layout, wells from the plates were pooled such that all A1 peptides were pooled, all A2 peptides, etc. maintaining the 96-well plate format, but reducing the overall number of samples that needed to be screened. 5 days post boost following transfection with hACE2 mRNA, splenocytes were harvested and stimulated with each peptide pool in the presence of brefeldin A. The frequency of IFN-γ+ CD4+ T cells is enumerated in a heat map format as the average responses of 3 mice. (TIF) [file ppat.1009163.s006.tif]

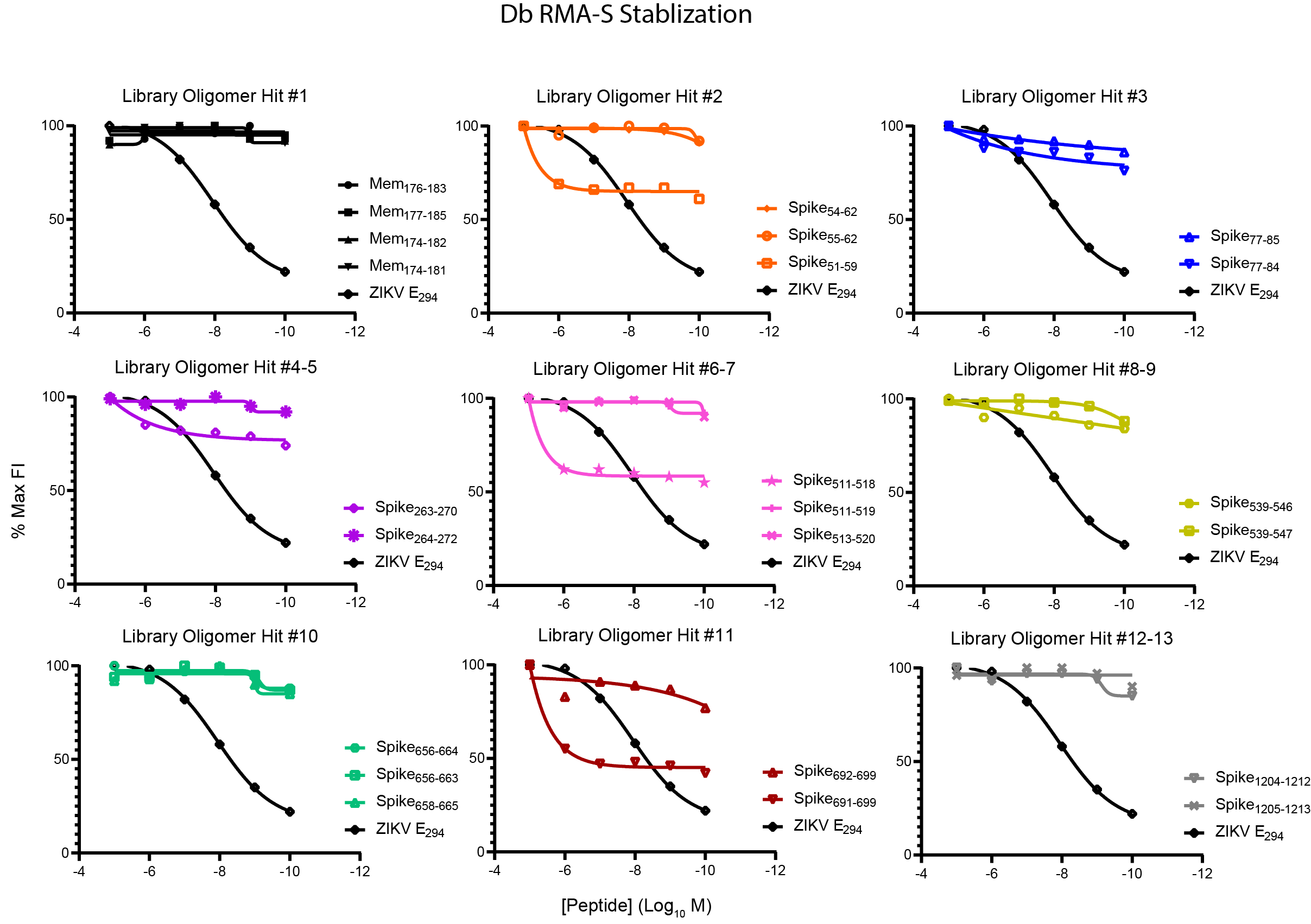

Supplement: S7 Fig — To determine relative ability of individual peptide variants to stabilize the Db molecule, decreasing concentrations of each peptide variant were incubated for 4 hours with TAP deficient RMA-S cells at 29 degrees C before being moved to 37 degrees C for 1 hour. Cells were then stained with anti- Db APC and geometric mean fluorescent intensity (gMFI) was measured on an Atttune focusing flow cytometer. Fluorescence index (FI) was determined by dividing the gMFI of cells pulsed with peptide by cells with no peptide. Data is presented as a percentage of the maximum FI for each peptide. As a positive control, the Db restricted peptide ZIKV E294 was used. (TIF) [file ppat.1009163.s007.tif]

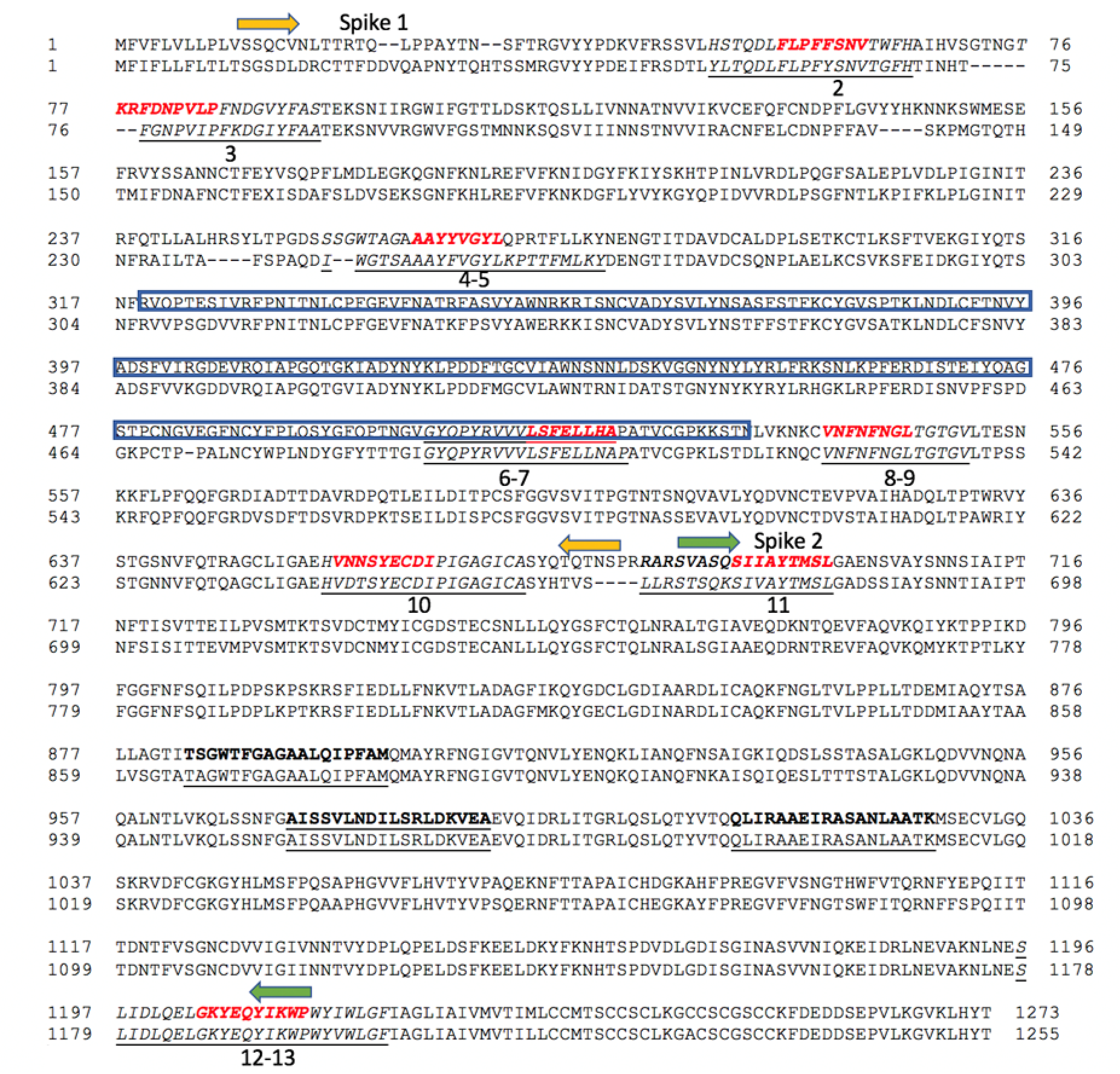

Supplement: S8 Fig — The amino acid sequences of SARS-CoV-2 Spike (QIG55857.1) and SARS-CoV Spike (ACZ72195.1) were aligned using NCBI based COBALT. Specific regions of interest included, the receptor binding domain (RBD) for SARS-CoV-2 is boxed blue, the location of SARS CoV-2 S1 of spike is noted by orange arrows and S2 is noted by green arrows. Underlined are the SARS-CoV CD4+ and CD8+ multi-mers that were initially screened in the epitope identification studies (Figs 3 and 4) and the numbers under each putative epitope are the panels that were screened for the final SARS-CoV-2 CD8+ T cell epitope identification. Bolded are the identified SARS-CoV-2 CD4+ T cell epitopes and in red italics are the identified SARS-CoV-2 CD8+ T cell epitopes. (TIF) [file ppat.1009163.s008.tif]

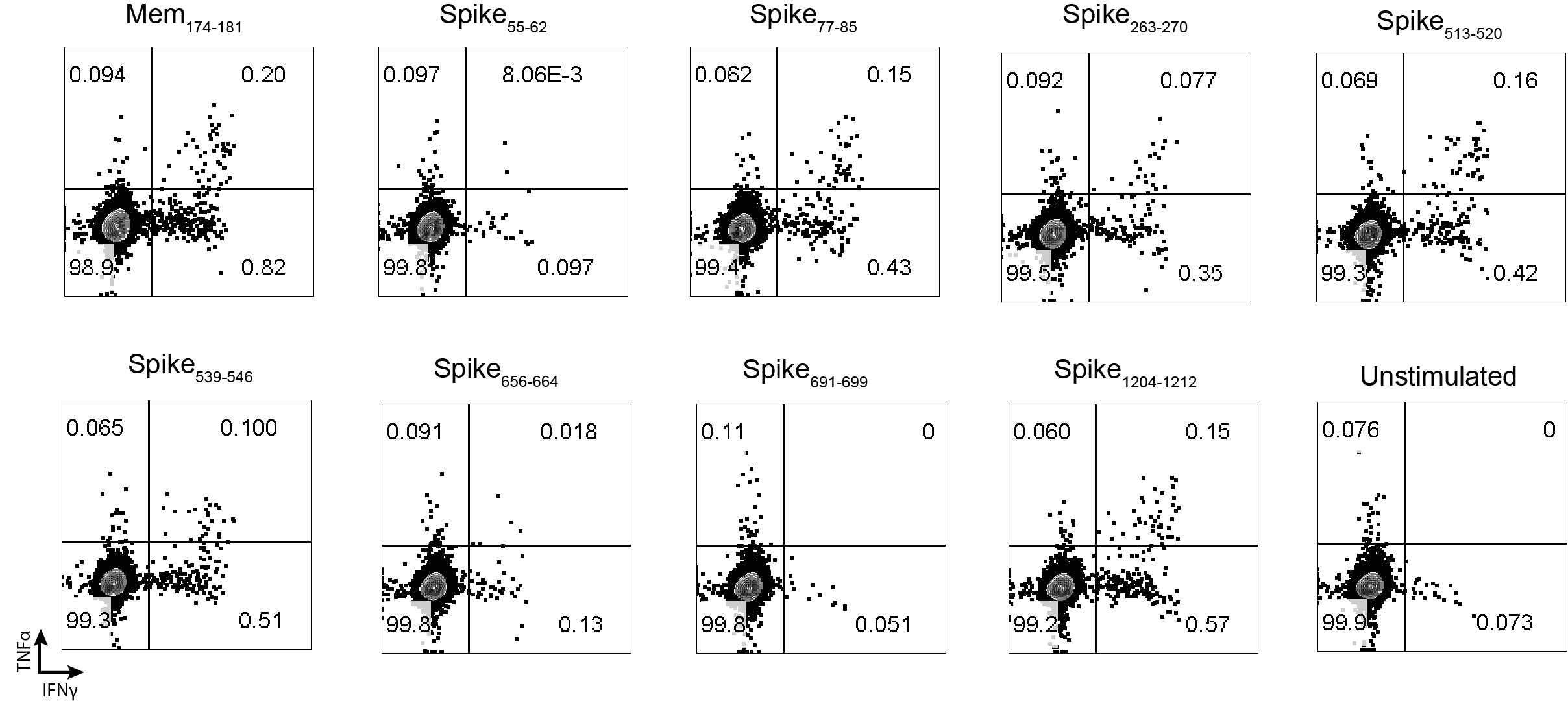

Supplement: S9 Fig — K18-hACE2 transgenic mice were infected with 104 FFU of SARS-CoV-2 (IN route). At day 10 post infection, a mouse was humanely euthanized and splenocytes harvested for peptide stimulation. Splenocytes were stimulated for 6 hours with each peptide in the presence of brefeldin A. After stimulation, cells were stained for flow cytometry to evaluate the frequency of responsive CD8+T cells by IFN-γ and TNF-α expression. (TIF) [file ppat.1009163.s009.tif]
