# Supplementary material for: mRNA induced expression of human angiotensin-converting enzyme 2 in mice for the study of the adaptive immune response to severe acute respiratory syndrome coronavirus 2
Source: PLoS Pathog. 2020 Dec 16;16(12):e1009163. doi: 10.1371/journal.ppat.1009163 (PMC7773324; doi:10.1371/journal.ppat.1009163)
Supplement: S1 Table — (PDF) [file ppat.1009163.s010.pdf]

**SARS-CoV S Protein Peptide Array (Catalog No. NR-2669)**

| Peptide | Amino acid sequence | Domain pool | Plate |
|---|---|---|---|
| 1 | MFIFLLFLTLTSGSDLDR | S1 N-Term | 1 |
| 2 | TLTSGSDLDRCTTFDDV | S1 N-Term | 1 |
| 3 | LDRCTTFDDVQAPNYTQH | S1 N-Term | 1 |
| 4 | DVQAPNYTQHTSSMRGVY | S1 N-Term | 1 |
| 5 | QHTSSMRGVYYPDEIFR | S1 N-Term | 1 |
| 6 | GVYYPDEIFRSDTLYL | S1 N-Term | 1 |
| 7 | EIFRSDTLYLTQDLFLPF | S1 N-Term | 1 |
| 8 | YLTQDLFLPFYSNVTGFH | S1 N-Term | 1 |
| 9 | PFYSNVTGFHTINHTF | S1 N-Term | 1 |
| 10 | TGFHTINHTFGNPVIPFK | S1 N-Term | 1 |
| 11 | TFGNPVIPFKDGIYFAA | S1 N-Term | 1 |
| 12 | PFKDGIYFAATEKSNVVR | S1 N-Term | 1 |
| 13 | AATEKSNVVRGWVFGSTM | S1 N-Term | 1 |
| 14 | VRGWVFGSTMNNKSQSVI | S1 N-Term | 1 |
| 15 | TMNNKSQSVIINNSTNV | S1 N-Term | 1 |
| 16 | VIINNSTNVVIRACNF | S1 N-Term | 1 |
| 17 | TNVVIRACNFELCDNPFF | S1 N-Term | 1 |
| 18 | NFELCDNPFFAVSKPM | S1 N-Term | 1 |
| 19 | NPFFAVSKPMGTQTHTMI | S1 N-Term | 1 |
| 20 | PMGTQTHTMIFDNAF | S1 N-Term | 1 |
| 21 | THTMIFDNAFNCTFEYI | S1 N-Term | 1 |
| 22 | NAFNCTFEYISDAFSLDV | S1 N-Term | 1 |
| 23 | YISDAFSLDVSEKSGNFK | S1 N-Term | 1 |
| 24 | DVSEKSGNFKHLREFVFK | S1 N-Term | 1 |
| 25 | FKHLREFVFKNKDGFLYV | S1 N-Term | 1 |
| 26 | FKNKDGFLYVYKGYQPI | S1 N-Term | 1 |
| 27 | LYVYKGYQPIDVVRDL | S1 N-Term | 1 |
| 28 | YQPIDVVRDLPSGFNTLK | S1 N-Term | 1 |
| 29 | DLPSGFNTLKPIFKLPL | S1 N-Term | 1 |
| 30 | TLKPIFKLPLGINITNFR | S1 N-Term | 1 |
| 31 | PLGINITNFRAILTAF | S1 N-Term | 1 |
| 32 | TNFRAILTAFSPAQDIW | S1 N-Term | 1 |
| 33 | TAFSPAQDIWGTSAAAYF | S1 N-Term | 1 |
| 34 | IWGTSAAAYFVGYLK | S1 N-Term | 1 |
| 35 | AAAYFVGYLKPTTFMLKY | S1 N-Term | 1 |
| 36 | LKPTTFMLKYDENGTI | S1 N-Term | 1 |
| 37 | MLKYDENGTITDAVDCSQ | S1 N-Term | 1 |
| 38 | TITDAVDCSQNPLAELK | S1 N-Term | 1 |
| 39 | CSQNPLAELKCSVKSFEI | S1 N-Term | 1 |
| 40 | LKCSVKSFEIDKGIY | S1 N-Term | 1 |
| 41 | KSFEIDKGIYQTSNFRVV | S1 N-Term | 1 |
| 42 | IYQTSNFRVVPSGDVVRF | RBD | 1 |
| 43 | VVPSGDVVRFPNITNL | RBD | 1 |
| 44 | VVRFPNITNLCPFGEVF | RBD | 1 |
| 45 | TNLCPFGEVFNATKFPSV | RBD | 1 |
| 46 | VFNATKFPSVYAWERKKI | RBD | 1 |
| 47 | SVYAWERKKISNCVADY | RBD | 1 |
| 48 | KKISNCVADYSVLYNSTF | RBD | 1 |
| 49 | DYSVLYNSTFFSTFKCY | RBD | 1 |
| 50 | STFFSTFKCYGVSATKL | RBD | 1 |
| 51 | KCYGVSATKLNDLCFSNV | RBD | 1 |
| 52 | KLNDLCFSNVYADSFVVK | RBD | 1 |
| 53 | NVYADSFVVKGDDVRQIA | RBD | 1 |
| 54 | VKGDDVRQIAPGQTGVIA | RBD | 1 |
| 55 | IAPGQTGVIADYNYKL | RBD | 1 |
| 56 | GVIADYNYKLPDDFMGCV | RBD | 1 |
| 57 | KLPDDFMGCVLAWNTRNI | RBD | 1 |
| 58 | CVLAWNTRNIDATSTGNY | RBD | 1 |
| 59 | NIDATSTGNYNYKYRYLR | RBD | 1 |
| 60 | NYNYKYRYLRHGKLRPF | RBD | 1 |
| 61 | YLRHGKLRPFERDISNV | RBD | 1 |
| 62 | RPFERDISNVPFSPDGK | RBD | 1 |
| 63 | SNVPFSPDGKPCTPPAL | RBD | 1 |
| 64 | DGKPCTPPALNCYWPL | RBD | 1 |
| 65 | PPALNCYWPLNDYGFY | RBD | 1 |
| 66 | YWPLNDYGFYTTTGIGY | RBD | 1 |
| 67 | GFYTTTGIGYQPYRVVVL | RBD | 1 |
| 68 | GYQPYRVVVLSFELLNA | RBD | 1 |
| 69 | VVLSFELLNAPATVCGPK | RBD | 1 |
| 70 | NAPATVCGPKLSTDLIK | RBD | 1 |
| 71 | GPKLSTDLIKNQCVNFNF | RBD | 1 |
| 72 | IKNQCVNFNFNGLTGTGV | RBD | 1 |
| 73 | NFNGLTGTGVLTPSSKRF | S1 C-Term | 1 |
| 74 | GVLTPSSKRFQPFQQFGR | S1 C-Term | 1 |
| 75 | RFQPFQQFGRDVSDF | S1 C-Term | 1 |
| 76 | QQFGRDVSDFTDSVRDPK | S1 C-Term | 1 |
| 77 | DFTDSVRDPKTSEILDI | S1 C-Term | 1 |
| 78 | DPKTSEILDISPCSFGGV | S1 C-Term | 1 |
| 79 | DISPCSFGGVSVITPGTNA | S1 C-Term | 1 |
| 80 | VSVITPGTNASSEVAVLY | S1 C-Term | 1 |
| 81 | NASSEVAVLYQDVNCTDV | S1 C-Term | 1 |
| 82 | LYQDVNCTDVSTAIHA | S1 C-Term | 1 |
| 83 | CTDVSTAIHADQLTPAWR | S1 C-Term | 1 |
| 84 | HADQLTPAWRIYSTGNNV | S1 C-Term | 1 |
| 85 | WRIYSTGNNVFQTQAGCL | S1 C-Term | 1 |
| 86 | NVFQTQAGCLIGAEHV | S1 C-Term | 1 |
| 87 | AGCLIGAEHVDTSYECDI | S1 C-Term | 1 |
| 88 | HVDTSYECDIPIGAGICA | S1 C-Term | 1 |
| 89 | DIPIGAGICASYHTVSLL | S1 C-Term | 1 |
| 90 | CASYHTVSLLRSTSQKSI | S1 C-Term | 1 |
| 91 | LLRSTSQKSIVAYTMSL | S2 | 1 |
| 92 | KSIVAYTMSLGADSSIAY | S2 | 1 |

| | | | |
|---|---|---|---|
| 93 | SLGADSSIAYSNNTIAI | S2 | 1 |
| 94 | IAYSNNTIAIPTNFSISI | S2 | 1 |
| 95 | AIPTNFSISITTEVMPV | S2 | 1 |
| 96 | ISITTEVMPVSMAKTSV | S2 | 1 |
| 1 | MPVSMAKTSVDCNMYI | S2 | 2 |
| 2 | KTSVDCNMYICGDSTECA | S2 | 2 |
| 3 | YICGDSTECANLLLQY | S2 | 2 |
| 4 | TECANLLLQYGSFCTQL | S2 | 2 |
| 5 | LQYGSFCTQLNRALSGIA | S2 | 2 |
| 6 | QLNRALSGIAAEQDRNTR | S2 | 2 |
| 7 | IAAEQDRNTREVFAQVK | S2 | 2 |
| 8 | NTREVFAQVKQMYKTPTL | S2 | 2 |
| 9 | VKQMYKTPTLKYFGGFNF | S2 | 2 |
| 10 | TLKYFGGFNFSQILPDPL | S2 | 2 |
| 11 | NFSQILPDPLKPTKRSFI | S2 | 2 |
| 12 | PLKPTKRSFIEDLLFNKV | S2 | 2 |
| 13 | FIEDLLFNKVTLADAGFM | S2 | 2 |
| 14 | KVTLADAGFMKQYGECL | S2 | 2 |
| 15 | GFMKQYGECLGDINARDL | S2 | 2 |
| 16 | CLGDINARDLICAQKF | S2 | 2 |
| 17 | ARDLICAQKFNGLTVL | S2 | 2 |
| 18 | AQKFNGLTVLPPLLTDDM | S2 | 2 |
| 19 | VLPPLLTDDMIAAYTAAL | S2 | 2 |
| 20 | DMIAAYTAALVSGTATA | S2 | 2 |
| 21 | AALVSGTATAGWTFGAGA | S2 | 2 |
| 22 | TAGWTFGAGAALQIPFAM | S2 | 2 |
| 23 | GAALQIPFAMQMAYRF | S2 | 2 |
| 24 | PFAMQMAYRFNGIGV | S2 | 2 |
| 25 | MAYRFNGIGVTQNVLY | S2 | 2 |
| 26 | GIGVTQNVLYENQKQIA | S2 | 2 |
| 27 | VLYENQKQIANQFNKAI | S2 | 2 |
| 28 | QIANQFNKAISQIQESL | S2 | 2 |
| 29 | KAISQIQESLTTTSTAL | S2 | 2 |
| 30 | ESLTTTSTALGKLQDVV | S2 | 2 |
| 31 | TALGKLQDVVNQNAQAL | S2 | 2 |
| 32 | DVVNQNAQALNTLVKQL | S2 | 2 |
| 33 | QALNTLVKQLSSNFGAI | S2 | 2 |
| 34 | KQLSSNFGAISSVLNDIL | S2 | 2 |
| 35 | AISSVLNDILSRLDKVEA | S2 | 2 |
| 36 | ILSRLDKVEAEVQIDRLI | S2 | 2 |
| 37 | EAEVQIDRLITGRLQSL | S2 | 2 |
| 38 | RLITGRLQSLQTYVTQQL | S2 | 2 |
| 39 | SLQTYVTQQLIRAAEIRA | S2 | 2 |
| 40 | QLIRAAEIRASANLAATK | S2 | 2 |
| 41 | RASANLAATKMSECVL | S2 | 2 |
| 42 | AATKMSECVLGQSKRVDF | S2 | 2 |
| 43 | VLGQSKRVDFCGKGYHLM | S2 | 2 |
| 44 | DFCGKGYHLMSFPQAAPH | S2 | 2 |

| | | | |
|---|---|---|---|
| 45 | LMSFPQAAPHGVVFLHV | S2 | 2 |
| 46 | APHGVVFLHVTYVPSQER | S2 | 2 |
| 47 | HVTYVPSQERNFTTAPAI | S2 | 2 |
| 48 | ERNFTTAPAICHEGKAYF | S2 | 2 |
| 49 | AICHEGKAYFPREGVFVF | S2 | 2 |
| 50 | YFPREGVFVFNGTSWFI | S2 | 2 |
| 51 | FVFNGTSWFITQRNFF | S2 | 2 |
| 52 | SWFITQRNFFSPQII | S2 | 2 |
| 53 | QRNFFSPQIITTDNTFV | S2 | 2 |
| 54 | QIITTDNTFVSGNCDVVI | S2 | 2 |
| 55 | FVSGNCDVVIGIINNTVY | S2 | 2 |
| 56 | VIGIINNTVYDPLQPEL | S2 | 2 |
| 57 | TVYDPLQPELDSFKEEL | S2 | 2 |
| 58 | PELDSFKEELDKYFKNH | S2 | 2 |
| 59 | EELDKYFKNHTSPDVDL | S2 | 2 |
| 60 | KNHTSPDVDLGDISGINA | S2 | 2 |
| 61 | DLGDISGINASVVNIQK | S2 | 2 |
| 62 | INASVVNIQKEIDRLNEV | S2 | 2 |
| 63 | QKEIDRLNEVAKNLNESL | S2 | 2 |
| 64 | EVAKNLNESLIDLQELGK | S2 | 2 |
| 65 | SLIDLQELGKYEQYIKW | S2 | 2 |
| 66 | LGKYEQYIKWPWYVWLGF | S2 | 2 |
| 67 | KWPWYVWLGFIAGLIAIV | S2 | 2 |
| 68 | GFIAGLIAIVMVTILL | S2 | 2 |
| 69 | IAIVMVTILLCCMTSCCSCL | S2 | 2 |
| 70 | CCMTSCCSCLKGACSCGS | S2 | 2 |
| 71 | CLKGACSCGSCCKFDEDD | S2 | 2 |
| 72 | GSCCKFDEDDSEPVLKGV | S2 | 2 |
| 73 | DDSEPVLKGVKLHYT | S2 | 2 |

| SARS-Related Coronavirus 2 Nucleocapsid (N) Protein Peptide Array, Catalog No. NR-52404 | | | |
|---|---|---|---|
| **Peptide** | **Amino acid sequence** | **Domain pool** | **Plate** |
| 1 | MSDNGPQNQRNAPRITF | NC N-Term | 3 |
| 2 | NQRNAPRITFGGPSDST | NC N-Term | 3 |
| 3 | ITFGGPSDSTGSNQNGE | NC N-Term | 3 |
| 4 | DSTGSNQNGERSGARSK | NC N-Term | 3 |
| 5 | NGERSGARSKQRRPQGL | NC N-Term | 3 |
| 6 | RSKQRRPQGLPNNTASW | NC RNA BD | 3 |
| 7 | QGLPNNTASWFTALTQH | NC RNA BD | 3 |
| 8 | ASWFTALTQHGKEDLKF | NC RNA BD | 3 |
| 9 | TQHGKEDLKFPRGQGVP | NC RNA BD | 3 |
| 10 | LKFPRGQGVPINTNSSP | NC RNA BD | 3 |
| 11 | GVPINTNSSPDDQIGYY | NC RNA BD | 3 |
| 12 | SSPDDQIGYYRRATRRI | NC RNA BD | 3 |
| 13 | GYYRRATRRIRGGDGKM | NC RNA BD | 3 |
| 14 | RRIRGGDGKMKDLSPRW | NC RNA BD | 3 |

| | | | |
|---|---|---|---|
| 15 | GKMKDLSPRWYFYYLGT | NC RNA BD | 3 |
| 16 | PRWYFYYLGTGPEAGLP | NC RNA BD | 3 |
| 17 | LGTGPEAGLPYGANKDG | NC RNA BD | 3 |
| 18 | GLPYGANKDGIIWVATE | NC RNA BD | 3 |
| 19 | KDGIIWVATEGALNTPK | NC RNA BD | 3 |
| 20 | ATEGALNTPKDHIGTRN | NC RNA BD | 3 |
| 21 | TPKDHIGTRNPANNAAI | NC RNA BD | 3 |
| 22 | TRNPANNAAIVLQLPQG | NC RNA BD | 3 |
| 23 | AAIVLQLPQGTTLPKGF | NC RNA BD | 3 |
| 24 | PQGTTLPKGFYAEGSRG | NC RNA BD | 3 |
| 25 | KGFYAEGSRGGSQASSR | NC RNA BD | 3 |
| 26 | SRGGSQASSRSSSRSRN | NC3 | 3 |
| 27 | SSRSSSRSRNSSRNSTP | NC3 | 3 |
| 28 | SRNSSRNSTPGSSRGTS | NC3 | 3 |
| 29 | STPGSSRGTSPARMAGN | NC3 | 3 |
| 30 | GTSPARMAGNGGDAALA | NC3 | 3 |
| 31 | AGNGGDAALALLLLDRL | NC3 | 3 |
| 32 | ALALLLLDRLNQLESKM | NC3 | 3 |
| 33 | DRLNQLESKMSGKGQQQ | NC3 | 3 |
| 34 | SKMSGKGQQQQGQTVTK | NC3 | 3 |
| 35 | QQQQGQTVTKKSAAEAS | NC3 | 3 |
| 36 | VTKKSAAEASKKPRQKR | NC3 | 3 |
| 37 | EASKKPRQKRTATKAYN | NC dimerization | 3 |
| 38 | QKRTATKAYNVTQAFGR | NC dimerization | 3 |
| 39 | AYNVTQAFGRRGPEQTQ | NC dimerization | 3 |
| 40 | FGRRGPEQTQGNFGDQE | NC dimerization | 3 |
| 41 | QTQGNFGDQELIRQGTD | NC dimerization | 3 |
| 42 | DQELIRQGTDYKHWPQI | NC dimerization | 3 |
| 43 | GTDYKHWPQIAQFAPSA | NC dimerization | 3 |
| 44 | PQIAQFAPSASAFFGMS | NC dimerization | 3 |
| 45 | PSASAFFGMSRIGMEVT | NC dimerization | 3 |
| 46 | GMSRIGMEVTPSGTWLT | NC dimerization | 3 |
| 47 | EVTPSGTWLTYTGAIKL | NC dimerization | 3 |
| 48 | WLTYTGAIKLDDKDPNF | NC dimerization | 3 |
| 49 | IKLDDKDPNFKDQVILL | NC dimerization | 3 |
| 50 | PNFKDQVILLNKHIDAY | NC dimerization | 3 |
| 51 | ILLNKHIDAYKTFPPTE | NC dimerization | 3 |
| 52 | DAYKTFPPTEPKKDKKK | NC C-Term | 3 |
| 53 | PTEPKKDKKKKADETQA | NC C-Term | 3 |
| 54 | KKKKADETQALPQRQKK | NC C-Term | 3 |
| 55 | TQALPQRQKKQQTVTLL | NC C-Term | 3 |
| 56 | QKKQQTVTLLPAADLDD | NC C-Term | 3 |
| 57 | TLLPAADLDDFSKQLQQ | NC C-Term | 3 |
| 58 | LDDFSKQLQQSMSSADS | NC C-Term | 3 |
| 59 | LQQSMSSADSTQA | NC C-Term | 3 |

| SARS-Related Coronavirus 2 Envelope (E) Protein Peptide Array, Catalog No. NR-52405 | | | |
|---|---|---|---|
| **Peptide** | **Amino acid sequence** | **Domain pool** | **Well pool** |
| 1 | MYSFVSEETGTLIVNSV | Env | 4 |
| 2 | ETGTLIVNSVLLFLAFV | Env | 4 |
| 3 | NSVLLFLAFVVFLLVTL | Env | 4 |
| 4 | AFVVFLLVTLAILTALR | Env | 4 |
| 5 | VTLAILTALRLCAYCCN | Env | 4 |
| 6 | ALRLCAYCCNIVNVSLV | Env | 4 |
| 7 | CCNIVNVSLVKPSFYVY | Env | 4 |
| 8 | SLVKPSFYVYSRVKNLN | Env | 4 |
| 9 | YVYSRVKNLNSSRVPDL | Env | 4 |
| 10 | NLNSSRVPDLLV | Env | 4 |

| SARS-Related Coronavirus 2 Membrane (M) Protein Peptide Array, Catalog No. NR-52403 | | | |
|---|---|---|---|
| **Peptide** | **Amino acid sequence** | **Domain pool** | **Well pool** |
| 1 | MADSNGTITVEELKKLL | Membrane | 5 |
| 2 | ITVEELKKLLEQWNLVI | Membrane | 5 |
| 3 | KLLEQWNLVIGFLFLTW | Membrane | 5 |
| 4 | LVIGFLFLTWICLLQFA | Membrane | 5 |
| 5 | LTWICLLQFAYANRNRF | Membrane | 5 |
| 6 | QFAYANRNRFLYIIKLI | Membrane | 5 |
| 7 | NRFLYIIKLIFLWLLWP | Membrane | 5 |
| 8 | KLIFLWLLWPVTLACFV | Membrane | 5 |
| 9 | LWPVTLACFVLAAVYRI | Membrane | 5 |
| 10 | CFVLAAVYRINWITGGI | Membrane | 5 |
| 11 | YRINWITGGIAIAMACL | Membrane | 5 |
| 12 | GGIAIAMACLVGLMWLS | Membrane | 5 |
| 13 | ACLVGLMWLSYFIASFR | Membrane | 5 |
| 14 | WLSYFIASFRLFARTRS | Membrane | 5 |
| 15 | SFRLFARTRSMWSFNPE | Membrane | 5 |
| 16 | TRSMWSFNPETNILLNV | Membrane | 5 |
| 17 | NPETNILLNVPLHGTIL | Membrane | 5 |
| 18 | LNVPLHGTILTRPLLES | Membrane | 5 |
| 19 | TILTRPLLESELVIGAV | Membrane | 5 |
| 20 | LESELVIGAVILRGHLR | Membrane | 5 |
| 21 | GAVILRGHLRIAGHHLG | Membrane | 5 |
| 22 | HLRIAGHHLGRCDIKDL | Membrane | 5 |
| 23 | HLGRCDIKDLPKEITVA | Membrane | 5 |
| 24 | KDLPKEITVATSRTLSY | Membrane | 5 |
| 25 | TVATSRTLSYYKLGASQ | Membrane | 5 |
| 26 | LSYYKLGASQRVAGDSG | Membrane | 5 |
| 27 | ASQRVAGDSGFAAYSRY | Membrane | 5 |
| 28 | DSGFAAYSRYRIGNYKL | Membrane | 5 |
| 29 | SRYRIGNYKLNTDHSSS | Membrane | 5 |
| 30 | YKLNTDHSSSDNIALL | Membrane | 5 |

| | | | |
|---|---|---|---|
| 31 | SSSSDNIALLVQ | Membrane | 5 |

Supplemental Table 1: Amino acid sequence information for the peptide libraries used in screens. Yellow highlights are the multi-mers that contain the identified CD8 T cell epitopes and the green highlights are the multi-mers that contain the identified CD4 T cell epitopes.
