# Supplementary material for: mRNA induced expression of human angiotensin-converting enzyme 2 in mice for the study of the adaptive immune response to severe acute respiratory syndrome coronavirus 2
Source: PLoS Pathog. 2020 Dec 16;16(12):e1009163. doi: 10.1371/journal.ppat.1009163 (PMC7773324; doi:10.1371/journal.ppat.1009163)
Supplement: Attachment — Submitted filename: Hassert- PLOS reviewer responses 11-10.docx [file ppat.1009163.s011.docx]

November 10, 2020

Shin-Ru Shih, PhD
Section Editor
*PLOS Pathogens*

Dear Dr. Shin-Ru Shih,

On November 3^rd^ we received the reviews for our manuscript entitled " mRNA induced expression of human angiotensin-converting enzyme 2 in mice for the study of the adaptive immune response to severe acute respiratory syndrome coronavirus 2" and we were invited to submit a revised version of the manuscript that addresses the issues and concerns raised by the reviews. We thank the reviewers for bringing up some excellent points in the previous study, and we thank you for inviting us to resubmit for consideration. We believe were able to address all of the two reviewers comments. This is a much better study due to the points that were brought up by the reviewers in the previous version and addressed by the laboratory in this revised version. Below we have listed each of the reviewer’s concerns and provided a point by point response in green. In addition to providing this response to the reviewer’s comments, we have also provided a clean revised manuscript and a marked-up copy of the changes made from the previous file, as you requested.

**Response to Reviewer’s Comments:**

**Part I - Summary**

Reviewer #1: The data presented in the current manuscript demonstrates there are obstacles in making this model a viable alternative to other methods. While in vitro characterization shows the ability of transiently transfected cells to express hACE2, this was not shown in vivo. The lack of good differentiating antibodies (between murine and human ACE2) is noted, but perhaps qPCR could have been performed in the nasal cavity or from lung homogenates to look for transgene expression. Infected hACE2-transfected IFNR KO mice did not induce significantly higher RBD-specific and neutralizing titers over control (GFP-transfected IFNR KO). This also holds true when looking for viral replication – virally-infected hACE2- and GFP-transfected IFNR KO mice did not demonstrate any significant differences. This suggests that the in vivo transfection may not have worked as successfully as anticipated – this should be addressed. The authors should try to figure the efficiency by which their mRNA administration in vivo is working. Lastly, while the authors were able to identify T-cell epitopes, that responding hACE2 mice had a large standard deviation, which suggest that transfection efficiency of the mRNA-ACE2 – however this is speculative on the reviewers part.

We thank the reviewer for their insightful and supportive comments. Since the submission and review of this manuscript an antibody that is able to distinguish between human Ace2 (hACE2) and murine ACE2 has become available so to address the reviewers concern/questions we have repeated the transfections and have added an additional figure 2 in the manuscript showing that: a) Mechanism of transfection we used in our studies results in detection of an unrelated protein (fLuc), b) following transfection the hACE2 mRNA is present at 24 hr post transfection in the lungs and liver and c) hACE2 transfection efficiency was approximately 15-20% as determined by protein expression by flow cytometry. In addition, we reworded portions of the introduction and conclusion to clarify the our murine model.

Line 123: It might be beneficial for the rest of the community if the authors list the antibodies that were used to attempt to differentiate human and murine ACE2 in the methods section. This can save others effort and funds.
We have added list of antibodies to methods. Additionally, we have since found an antibody that works and this is also included in the methods

Line 151: It would be useful to know the concentration or the amount of mRNA that was used for each route of administration (intravenous and intranasal). Is there a rationale why both administrations were utilized instead of one or the other. Our own experience with using the Polyplus in vivo-jet RNA transfection system suggests that transgene expression via the intranasal route was inferior (metric was protection against a lethal viral challenge) to a systemic intramuscular route of administration. Have the authors administered the mRNA either intranasal only or intramuscular only and compare the efficiency of gene expression? It may benefit the readers to know whether one route of administration is sufficient instead of using both routes.

We thank the reviewer for their insightful comment this information can be found with the methods section for the mRNA transfection. The amount is (10ug). To aid in clarifying the concentration and with the inclusion of figure 2 the method section has been expanded highlighting the amount transfected. As our mRNA system is being used for transfection with the goal of expression of hACE2bin the lungs rather than vaccination an intramuscular route of administration would not be ideal.

Line 369: The authors should also include in the discussion the possibility that the intranasal administration of mRNA was not efficient in delivering the mRNA encoding hACE2. Have the authors assessed the optimal amount of RNA delivered? This and the points discussed by the authors may contribute to not detecting any measurable viral replication or pathogenesis in the current experimental model. Lastly, the results from this study demonstrates that there is a robust B- and T-cell response to SARS-CoV-2 infection –but it could be reflective of the route of RNA administration that is most efficient in delivery (IV vs IN). This is speculative; however, this may help explain the current results.

We thank the reviewer for their helpful comments, as in this study we were aiming to express hACE2 in the lungs of animals to make them more susceptible to SARS-CoV-2 we in delivering 10ug of mRNA we were within the manufacturer’s suggested amounts however we agree that it is always possible that more mRNA could results in more hACE2 expression. As the newly added figure 2 demonstrates the delivery of 10ug of mRNA did result in detectible hACE-2 expression and this amount did allow our group to study the adaptive immune responses reported in this manuscript. Finally we agree with the reviewer the route of administration of the hACE2 mRNA (IV and IN) contributed to the ability of SARS CoV-2 to induce an adaptive immune response.

Reviewer #2: Using an mRNA delivery platform to induce expression of hACE2 in mice, Hassert et al. investigate the adaptive immune response to SARS-CoV-2 in type I interferon deficient mice. They demonstrate that high affinity antibody response to SARS-CoV-2 is independent of the hACE2 expression, but they found a more robust T cell response when hACE2 mRNA was provided to mice. The authors further profile and define CD8+ and CD4+ structural peptide epitopes that are targeted during infection, and find similar targets as to what is seen in humans.

Unfortunately, beyond an indirect T cell response, there is no evidence that this mouse model system induced infection. The paper as framed suggests otherwise. Further, there is no evidence of mRNA expression of hACE2 in the actual mouse model, the paper also suggests otherwise. Validation of mRNA expression was done in cell culture not in vivo.

We thank the reviewer for their insights, to address their concerns and demonstrate expression of hACE2 by mRNA transfection in vivo we have included an additional figure in the manuscript, figure 2. Figure 2 demonstrates a) Mechanism of transfection results in detection of an unrelated protein (fLuc), b) mRNA is present at 24 hr post transfection in the lungs and liver and c) hACE2 transfection efficiency was approximately 15-20% depending on the animal. In addition, we reworded to reflect the use of this model for exploring the adaptive response to SARS-CoV-2.

**Part II – Major Issues: Key Experiments Required for Acceptance**

Reviewer #1: The authors should show the efficiency by which the mRNA is being delivered intranasally or systemically. This will allow them to figure out the viability of their model being susceptible to SARS-CoV-2 infection or immunization.

To address this criticism we have added an additional figure, figure 2 which demonstrates that both with qPCR and flow cytometry in the lungs and liver 24 hours post transfection that we are able to see stable expression of hACE-2b with a transfection efficiency of ~20% at the time of infection.

Reviewer #2: Unfortunately, beyond an indirect T cell response, there is no evidence that this mouse model system induced infection. The paper as framed suggests otherwise. Further, there is no evidence of delievered mRNA expression of hACE2 in the actual mouse model, the paper also suggests otherwise. Validation of mRNA expression was done in cell culture not in vivo.

Noted above in response to the summary comments, in the newly added figure 2 we have shown that the with the amount of mRNA delivered we were able to 1) show that the hACE2 was present 24 hours after transfection, which was the time of SARS-CoV-2 administration and 2) that at 24 hours we were able to achieve between a 15 and 20 percent transfection efficiency of our mRNA expressing hACE2 in the lungs of the transfected mice. 3) Additionally, included in the newly presented figure 2 we have shown via whole mouse imagining (IVIS) the mRNA expression of an unrelated protein fLuc 24 hours following transfection using a similar dose and route as we used for the hACE2 mRNA transfection.

**Part III – Minor Issues: Editorial and Data Presentation Modifications**

Reviewer #1: (No Response)

Reviewer #2:

1) Figure 1C and 1D have no error bars? Since infection initiation only probed at 24H, expression levels should be assessed at 24H.
We have added additional data to figure 1 which allowed the inclusion of error bars.

2) No significant differences in viral loads in mice or any infectious virus found, yet genome copies were found in all organs. Authors should use non-infected control mouse group to confirm primer specificity.

The primers used in these studies are the standard primers

We ran qPCR on lungs and livers of uninfected mice and did not detect any viral transcripts all the samples were not detectable.

3) Robust T cell response compared to what? Need positive control here (ie transgenic hACE2 mouse).

The robust T cell responses were compared to the GFP control which did not show a T cell response following infection. Additionally as we have hACE2 transgenic mice available we have included them a s a positive control, by adding supplemental figure 9, which demonstrates T cell responses in K18 mice.

We appreciate the opportunity to publish in *PLOS Pathogens*, and would like to thank you and the reviewers for the, insightful, and positive feedback on our manuscript. We hope this revised manuscript is acceptable for publication in *PLOS Pathogens*. Please do not hesitate to contact me for any reason.

Sincerely,

Amelia Pinto Ph.D.

Assistant Professor

Saint Louis University- School of Medicine

Department of Molecular Microbiology and Immunology

Doisy Research Center

1100 S. Grand Blvd., 703

St. Louis, MO 63104

[amelia.pinto@health.slu.edu](mailto:amelia.pinto@health.slu.edu)
